# A novel mutation in PCD‐associated gene *DNAAF3* causes male infertility due to asthenozoospermia

**DOI:** 10.1111/jcmm.17881

**Published:** 2023-08-03

**Authors:** Feng Wan, Lan Yu, Xiaowei Qu, Yanqing Xia, Ke Feng, Lei Zhang, Na Zhang, Guihua Zhao, Cuilian Zhang, Haibin Guo

**Affiliations:** ^1^ The Reproductive Medicine Center Henan Provincial People's Hospital Zhengzhou China; ^2^ The Reproductive Medicine Center People's Hospital of Zhengzhou University Zhengzhou China; ^3^ The Reproductive Medicine Center Henan Provincial People's Hospital of Henan University Zhengzhou China; ^4^ Department of Cardiopulmonary Function Henan Provincial People's Hospital Zhengzhou China

**Keywords:** asthenozoospermia, *DNAAF3*, male infertility, PCD, whole‐exome sequencing

## Abstract

Primary ciliary dyskinesia (PCD) is a rare autosomal‐recessive disease manifested with recurrent infections of respiratory tract and infertility. *DNAAF3* is identified as a novel gene associated with PCD and different mutations in *DNAAF3* results in different clinical features of PCD patients, such as situs inversus, sinusitis and bronchiectasis. However, the sperm phenotypic characteristics of PCD males are generally poorly investigated. Our reproductive medicine centre received a case of PCD patient with infertility, who presented with sinusitis, recurrent infections of the lower airway and severe asthenozoospermia; However, no situs inversus was found in the patient. A novel homozygous mutation in *DNAAF3*(c.551T>A; p.V184E) was identified in the PCD patient by whole‐exome sequencing. Subsequent Sanger sequencing further confirmed that the *DNAAF3* had a homozygous missense variant in the fifth exon. Transmission electron microscopy and immunostaining analysis of the sperms from the patient showed a complete absence of outer dynein arms and partial absence of inner dynein arms, which resulted in the reduction in sperm motility. However, this infertility was overcome by intracytoplasmic sperm injections, as his wife achieved successful pregnancy. These findings showed that the PCD‐associated pathogenic mutation within *DNAAF3* also causes severe asthenozoospermia and male infertility ultimately due to sperm flagella axoneme defect in humans. Our study not only contributes to understand the sperm phenotypic characteristics of patients with *DNAAF3* mutations but also expands the spectrum of *DNAAF3* mutations and may contribute to the genetic diagnosis and therapy for infertile patient with PCD.

## 1 INTRODUCTION

Infertility is becoming a major public medical and social issue.^1^ Approximately 10%–15% of couples worldwide are confronted with infertility and 50% of the cases are attributed to male factors.^2^, ^3^ Approximately 7% of all men suffer from infertility worldwide.^4^ Asthenozoospermia is one of the common causes of male infertility, which is involved in more than 40% the cases.^5^, ^6^ Based on the WHO ‘Manual Semen Inspection and Handling Laboratory Manual’ (fifth edition) criteria, asthenozoospermia is divided into the following four types: (i) mild asthenozoospermia; (ii) moderate asthenozoospermia; (iii) severe asthenozoospermia; and (iv) complete asthenozoospermia, according to the total motility of sperms.^7^, ^8^ Due to the reduction or the absence of motile sperms, severe asthenozoospermia and complete asthenozoospermia become a challenge for reproductive medicine. Previous studies have revealed that genetic factors may be the major underlying cause for severe asthenozoospermia. However, the exact pathogenesis of asthenozoospermia is unclear in most cases.

Sperm flagella and motile cilia share an evolutionarily conserved axonemal structure, which comprises nine doublets of microtubules circularly arranged around a central pair of singlet microtubules, a typical ‘9 + 2’ axonemal structure.^9^ Among the intricate axonemal protein complexes, the outer and inner dynein arms (ODAs and IDAs, respectively), along with each outer doublet microtubules, play a major role in the beating of both sperm flagella and cilia through hydrolyse ATP to power flagellar and ciliary movement.^10^, ^11^, ^12^ Moreover, dynein assembly factors are essential for the cytoplasmic assembly of dynein arms preceding their import into the axoneme. Because of the highly conservative and structural similarities between motile cilia and flagella, the axoneme defects not only lead to primary ciliary dyskinesia (PCD), a genetic disease characterized by chronic respiratory tract infections, but also male infertility due to asthenozoospermia.^13^, ^14^, ^15^ PCD is a rare, autosomal‐recessive disease and is genetically heterogeneous because of various of ultrastructural ciliary axoneme defects, and more than 70% of the PCD‐affected individuals lose the ODAs and IDAs.^16^, ^17^, ^18^, ^19^ Though gene mutations that causing PCD have been reported to be associated with male infertility, such as *DNAH5* ^20^ *DNAI1*,^21^ *DNAI2* ^22^ and *DNAH9*,^23^ which encode ODA components, the sperm phenotype of PCD patients is generally poorly investigated. In addition, PCD‐causing mutations in genes encoding dynein assembly factor have been identified, such as *DNAAF1*/*DNAAF2* mutations that fails to preassembly of dynein arm complexes in the cytoplasm.^24^, ^25^, ^26^ Dynein axonemal assembly factor 3 (*DNAAF3*) is identified as a novel gene associated with PCD, which is located on human chromosome 19q13 and encodes a 588 amino acids protein (GeneBank ID: NP_849159). Previous studies showed that *DNAAF3* mutations in PCD patients cause deficiency or loss of outer and inner dynein arms. Zebrafish *dnaaf3* knockdown causes PCD phenotypes due to immotile cilia with dynein arm assembly defects.^27^ However, whether *DNAAF3* mutation in PCD patients affect sperm motility due to loss of the flagella dynein arms is unknown. Here, we identified a novel mutation in *DNAAF3* from an infertile and PCD patient with severe asthenozoospermia and revealed that the deleterious mutation of *DNAAF3* may be the key factor responsible for infertility.

## 2 METHODS

### 2.1 Patients

PCD patient with infertility and his family were recruited from Henan Provincial People's Hospital, Henan Provincial Reproductive Hospital. This study was approved by the Ethics Committee of Henan Provincial Reproductive Hospital. Written informed consent was obtained, and 5 mL of peripheral blood was collected from all family members participating in this study.

### 2.2 Radiological examination

Radiological examinations were taken to detect the effect of *DNAAF3* mutation on respiratory system. High‐resolution computed tomography (HRCT) (SIMENS SOMATOM Definition Flash CT, German) of chest and paranasal sinus was performed to detect the pathological changes in the respiratory system and paranasal sinus.

### 2.3 Whole‐exome sequencing

Whole‐exome sequencing (WES) was performed to detect gene mutations. Briefly, genomic DNA (gDNA) was obtained from peripheral blood using the QIAamp DNA Blood Mini Kit according to the manufacturer's instructions. Agarose gel electrophoresis and Qubit were used to detect DNA integrity, concentration and purity. The isolated DNAs were fragmented using Covaris S220 focused ultrasonication, and then, the library construction was performed using the NEBNext Ultra II DNA Library Prep Kit for Illumina kit (NEB) for interrupted DNA. Library fragment length was determined using an Agilent 2100 Bioanalyzer system (Agilent DNA 1000 Kit), and coding regions and intron/exon boundaries were enriched using the TruSeq Exome Enrichment Kit (Illumina). DNA sequencing was undertaken using the Truseq SBS Kit V4‐HS kit on an NextSeq 550DX (Illumina) platform. Sequence reads were aligned to the reference genome (GRCh37/hg19) using Burrows–Wheeler Aligner (http://biobwa.sourc eforge.net/). Single nucleotide variants and small insertions/deletions (InDels) were identified and quality‐filtered using SAMtools (http://samtools.sourceforge.net/). Variants were annotated by the ANNOVAR (http://www.openbioinformat ics.org/annovar/) database, the Exome Aggregation Consortium (ExAC) (http://exac.broadinstitute.org/), Mutation Taster (http://www.mutationtaster.org/) database and the 1000 Genomes project (http://www.1000genomes.org/data) databases for pathopoiesia, novelty and frequency. Based on Ensembl (http://www.ensembl.org), the detected variants with a minor allele frequency greater than 1% in 1000 Genomes Project (ftp://ftp.1000genomes.ebi.ac.uk/vol1/ftp) or ExAC (http://exac.broadinstitute.org/) were discarded. Variants causing frameshift, missense, nonsense or impacting splice sites were retained for subsequent analyses. SIFT^28^ and Polyphen‐2^29^ were used to predict the impact of missense variants.

### 2.4 Sanger sequencing validation

To validate the mutation of the *DNAAF3* gene in the patient and his family, conventional Sanger sequencing (the primers: *DNAAF3* F 5′‐ATTCCCTCTTCTAGGTGGTTATTT‐3′, R 5′‐GACTGTGCGCTTCCATTATTC‐3′) was carried out. Primers were designed based on the Human Genome Sequence (GRCh38/hg19) and the reference sequence NM_001256714 of *DNAAF3* gene.

### 2.5 Immunostaining of spermatozoa

Immunofluorescence staining of spermatozoa was performed from control individual and the patient carrying *DNAAF3* mutation. Spermatozoa were smeared onto 0.1% poly L‐lysine pre‐coated glass slides and fixed with 4% paraformaldehyde in phosphate‐buffered saline (PBS) for 10 min at room temperature. After washing with PBS, spermatozoa were permeabilized and blocked using 0.2% Triton X‐100–DPBS (Triton X‐100; Sigma‐Aldrich) with 5% normal serum‐DPBS (Bull Serum Albumin; GIBCO, Invitrogen) for an hour. Then, the spermatozoa were incubated with the following primary antibodies: rabbit polyclonal anti‐DNAH5 (HPA037470, Sigma‐Aldrich, 1:100), rabbit polyclonal anti‐DNALI1 (HPA028305, Sigma‐Aldrich, 1:100) and monoclonal mouse anti‐acetylated‐a‐tubulin (T7451, Sigma‐Aldrich, 1:2000) overnight at 4°C and followed by secondary antibodies for an hour at room temperature. Spermatozoa were counterstained with DAPI (blue) as a nuclei marker. Fluorescence images were captured with a confocal microscope (Zeiss LSM 710).

### 2.6 Transmission electron microscopy

Transmission electron microscopy (TEM) was performed to examine the subcellular structural changes in sperm according to standard protocols. Briefly, spermatozoa were fixed with 2.5% phosphate‐buffered glutaraldehyde. Spermatozoa were then washed with 0.1 M phosphate buffer (pH 7.4) three times, postfixed with 1% osmium tetroxide, dehydrated in an ethanol series, infiltrated with 1:1 acetone and SPI‐CHEM resin, embedded and polymerized. Ultrathin 70‐nm thick sections were cut and were then collected on copper grids, stained with aqueous uranyl‐acetate and lead citrate. The images of spermatozoa ultrastructure were obtained using a JEM‐1400 (JAPAN) transmission electron microscope at 80 kV.

### 2.7 In vitro functional experiment

In order to study the effects of the mutation (c.551T>A; p.V184E) on the protein of DNAAF3, we performed a transient transfection assay with *DNAAF3* mutation plasmids. The full‐length coding sequence of the wild‐type (WT) *DNAAF3* and mutant‐type (MUT) *DNAAF3* sequence (c.551T>A) were synthesized and were then cloned these sequences into EGFP‐N1 overexpression vector by restriction enzyme digestion and ligation using CutSmart® Buffer (B7204S, New England Biolabs Inc.). Thus, DNAAF3 was fused with the EGFP tag for expression in vitro and the detection of EGFP protein reflects the expression of DNAAF3 protein. Then, the constructed EGFP‐N1‐*DNAAF3*‐WT and EGFP‐N1‐*DNAAF3*‐MUT plasmids were transfected into HEK‐293T cells using a lipofectamine 2000 transfection kit (Invitrogen). The HEK‐293T cells transfected lipofectamine used as negative control group (NC). In Brief, Lipofectamine 2000 reagent and plasmids were separately diluted by Opti‐MEM medium and then mixed and incubated for 10–15 min at room temperature. The plasmid–lipid complex was transfected to 50%–70% confluent HEK‐293T cells in a 6‐well culture dish. Twenty‐four hours after transfection, the plasmid expression was observed under a OLYMPUS IX73 fluorescence microscope at 100× magnification. Then, 72 h after transfection, the transfected cells were collected to examine DNAAF3 expression according to the standard western blotting procedures.

### 2.8 Western blot assay

Western blot assay was used to detect the change in DNAAF3 expression in the HEK‐293T cells transfected with EGFP‐N1‐*DNAAF3*‐WT and EGFP‐N1‐*DNAAF3*‐MUT (c.551T>A; p.V184E) plasmids. The total protein from cells was extracted using RIPA Buffer (P0013B, Beyotime Biotechnology) according to the manufacturer's instructions. Equal amounts of protein samples were resolved on a 10% sodium dodecyl sulphate‐polyacrylamide gel electrophoresis (SDS‐PAGE) and transferred to nitrocellulose membranes (Millipore Corporation). The membranes were incubated with the primary antibody GFP (ab290, Abcam, 1:2000). GAPDH (ab8245, Abcam, 1:1000) was used as the internal control. After incubation with HRP‐labelled secondary antibody, the immunoreactive bands were visualized by ultra‐sensitive ECL reagent (P0018, Beyotime Biotechnology) and the intensity of the detected bands was analysed using an Image J program and normalized to the corresponding GAPDH. The data obtained in experiments are presented as mean ± SD, and the differences between two groups were counted by two independent samples *t*‐test. The data were considered significant when the *p*‐value was less than 0.05 (*).

### 2.9 Intracytoplasmic sperm injection and embryo culture

Intracytoplasmic sperm injection (ICSI) was carried out in the patient with asthenozoospermia. Briefly, the ejaculated sperm of the PCD‐associated patient was obtained after centrifuged at 400 *g* for 10 min and resuspended to 150 μL of medium. The hypo‐osmotic swelling test (HOST) is used to select immotile spermatozoa prior to ICSI. Eggs were retrieved after the long‐acting GnRHa protocol followed by injection of HCG. ICSI trial was performed on 15 April 2019, and the embryos were cultured using Vitrolife G‐SERIES culture media (Vitrolife). The good‐quality embryos were selected for frozen and were then transferred 2 months later.

## 3 RESULTS

### 3.1 Patients and family

The proband, 28‐year‐old man, was diagnosed as a primary infertility a year ago due to asthenozoospermia according to the WHO ‘Manual Semen Inspection and Handling Laboratory Manual’ (fifth edition) criteria. Asthenozoospermia was diagnosed when his total motility (progressive motility and nonprogressive motility) is less than 40% or when his progressive motility is below 32% based on the ejaculate sperm motility. The proband and his consanguineous families were recruited from Henan Provincial People's Hospital, Henan Provincial Reproductive Hospital. The patient, with no history of smoking, has suffered from chronic airway infections and rhinosinusitis for 28 years and has a history of sinusitis resection. The patient has a normal somatic karyotype (46, XY) and no Y chromosome microdeletions. In addition, the development of genital organs, the seminal plasma biochemical indexes and the reproductive hormones were normal. The sperm morphology and the semen examination parameters were also normal except for reduced sperm motility. The percentage of progressive sperm was 1.7% and the percentage of nonprogressive sperm was 0.49%. The sperm viability was 46% with hypoosmotic swelling test (HOST) (%). CT tomogram showed no situs inversus. The specific clinical manifestations of the proband and his sister are shown in Table 1.

**TABLE 1:** Summary of the clinical features of the PCD family (II:1‐II:3).

Characteristics	II:1	II:3
Gender	Male	Female
Age	28 years	31 years
Birth status	Full‐term natural labour with a weight of 3.2 kg	Full‐term natural labour with a weight of 2.8 kg
Onset	Cough, expectoration and fever, the second month of birth	NO
Respiratory symptom	Severe respiratory symptoms: Bronchitis, intermittent wet cough, fever, nasal congestion, and difficulty breathing	NO
Situs inversus	NO	NO
Otitis media	NO	NO
Sinusitis	YES	NO
Age of diagnosis of bronchial asthma	12 year	NO
History of respiratory surgery	Partial resection of the left lung was performed due to pulmonary abscess at the age of 12 years; the nasal polyps resection was treated at the age of 14 years	NO
Fertility problems	Primary infertility for 3 years	NO
Smell problems	NO	NO
Hearing problems	NO	NO
History of dust, smoking or toxicant exposure	NO	NO
Respiratory function test	Severe mixed ventilatory dysfunction with reduced small airway function, but normal diffuse function	Unknown
FeNO	No abnormality	Unknown
FEV1/FVC	54.67%	Unknown
FEV1	1.89	Unknown

Abbreviations: FeNO, fractional exhaled nitric oxide; FEV1, forced expiratory volume in 1 s; FVC, forced vital capacity.

### 3.2 Radiological examination

**FIGURE 1:**
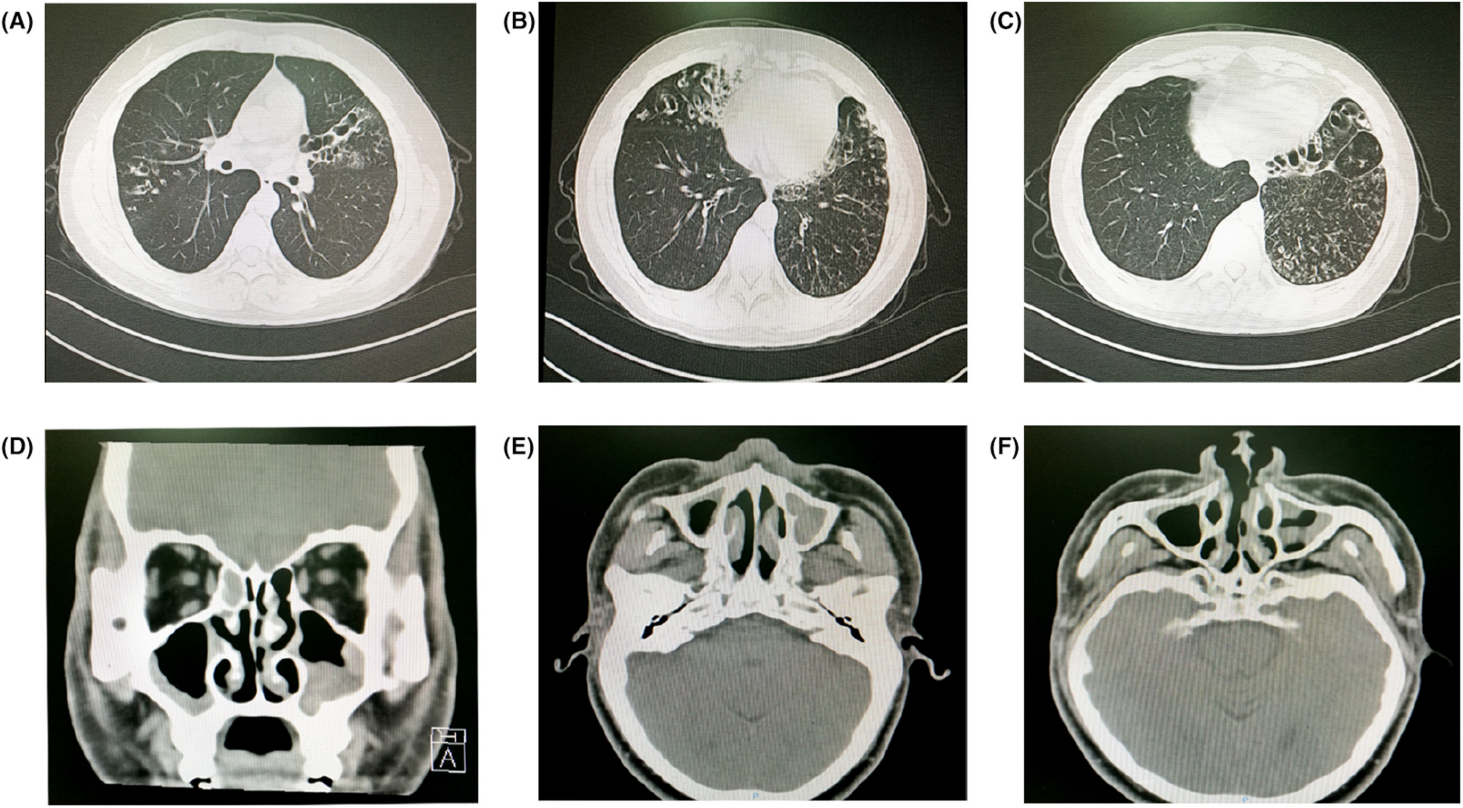
Radiological examination of proband (II:1). (A–C) Chest HRCT images showing multiple cystic bronchiectasis and bronchial wall thickening, and infection sign, such as multiple spot‐like and sheet‐like increasing density images are seen in both lung lobes; scattered cable‐like shadows are seen in both lung lobes suggest previous infection. (D) Coronal view of paranasal sinus HRCT showing bilateral maxillary sinusitis and ethmoid sinusitis. (E and F) Transverse view of sinus HRCT showed bilateral maxillary sinusitis and sphenoid sinus.

Chest HRCT of the proband showed multiple cystic bronchiectasis and bronchial wall thickening, multiple spot‐like and sheet‐like increasing density images and scattered cable‐like shadows in both lung lobes, which indicate bilateral bronchiectasis with infection. Paranasal sinus HRCT showed mucosal thickening in the bilateral maxillary sinus, ethmoid sinus and sphenoid sinus, which indicates sinusitis (Figure 1A–F). Based on these results and the following *DNAAF3* gene mutation validation, PCD along with asthenozoospermia was diagnosed.

### 3.3 The ultrastructure defects of the sperm were identified in the PCD patient with severe asthenozoospermia by transmission electron microscopy analysis

**FIGURE 2:**
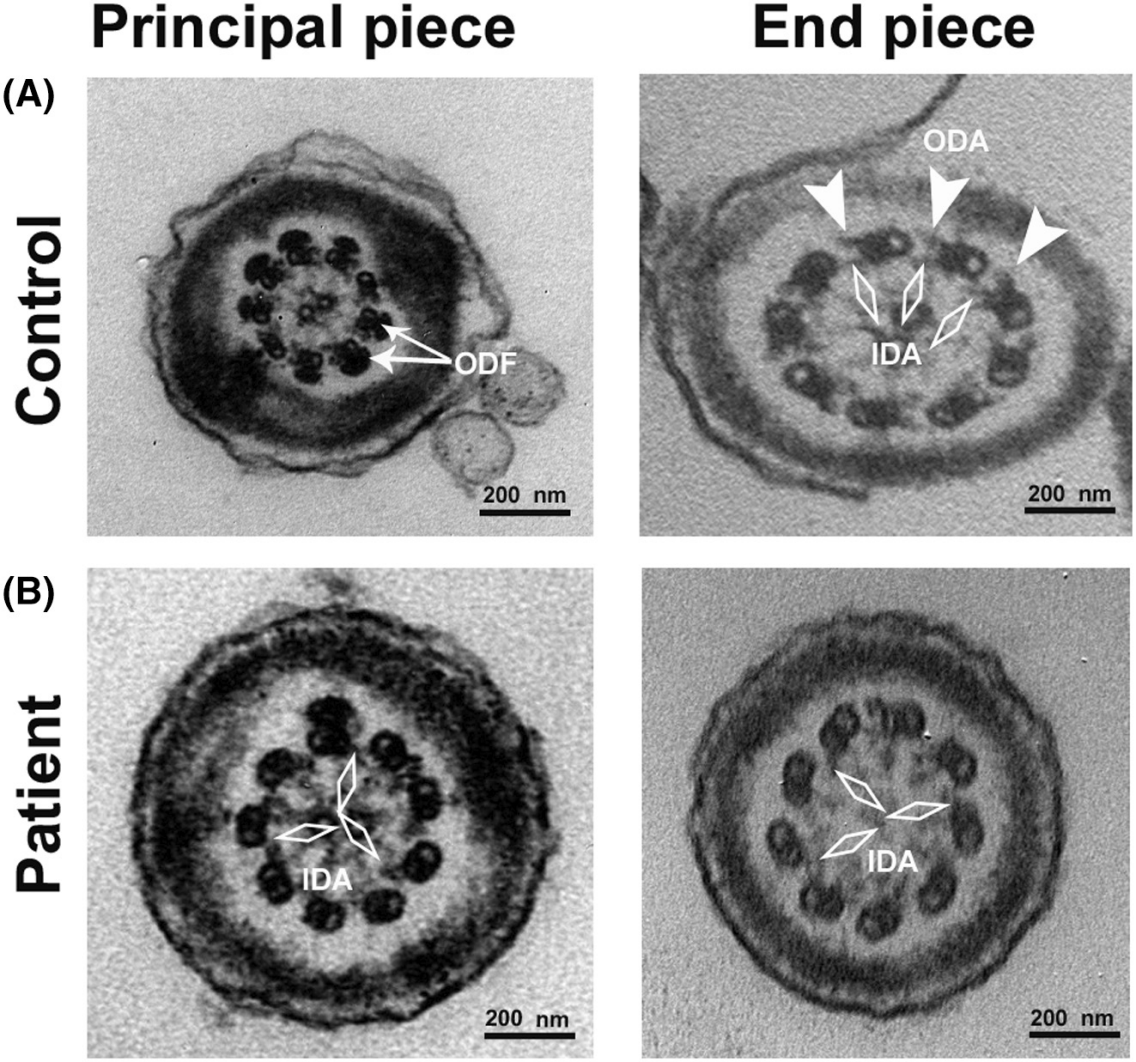
Transmission electron microscope (TEM) analyses of spermatozoa from a control individual and the proband (II:1). (A) Cross‐section of principal piece and end piece of the control flagellum showing normal outer dense fibres (ODF), outer dynein arms (ODAs) and inner dynein arms (IDAs). Outer dynein arms (ODAs) are indicated by white triangles and inner dynein arms (IDAs) are indicated by white rhombus in the control. (B) Cross‐section of principal piece and end piece of the proband flagellum showing complete absence of ODAs and partial absence IDAs. The absent ODAs and IDAs are indicated by white triangles and white rhombus respectively.

Previous studies showed that DNAAF3 was indispensable for assembly of axonemal inner and outer dynein arms. The inner and outer dynein arms assembly are deficient in motile cilia of the PCD patients carrying *DNAAF3* mutations. To investigate whether *DNAAF3* mutation affects the sperms of the PCD patient, TEM was performed to detect the ultrastructure of axoneme. The normal spermatozoon from the control showed the typical ‘9 + 2’ microtubule structure. The outer and inner dynein arms, and few other periaxonemal structures, such as the outer dense fibres (ODF) were well organized (Figure 2A). As indicated by the black arrow (Figure 2B), the spermatozoa from the PCD patient showed a complete absence of outer dynein arms and partial absence of inner dynein arms in the cross‐section of principal piece and end piece.

### 3.4 A novel mutation of the *DNAAF3* gene was identified in the PCD patient with severe asthenozoospermia

**FIGURE 3:**
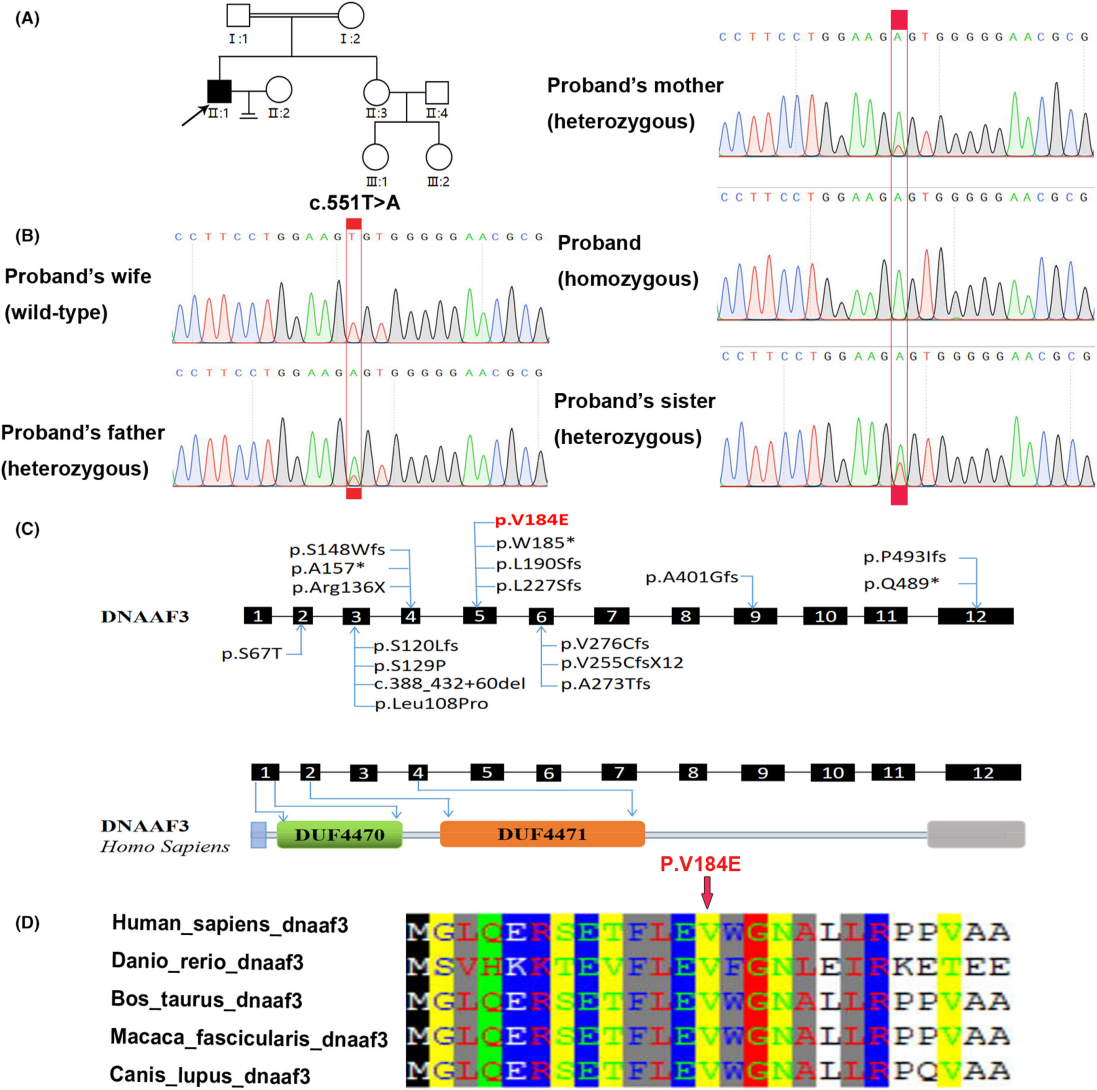
Identification of *DNAAF3* mutations in the proband (II:1) from a consanguineous family. (A) Pedigree of the consanguineous family with Primary ciliary dyskinesia and infertility. The double line indicates consanguinity between parents. The primary ciliary dyskinesia patient with infertility is indicated with shading, Squares and circles denoted males and females, respectively. (B) Sanger sequencing data of the mutation *DNAAF3* identified in the family: c.551T>A; p.V184E. The PCD‐affected proband with infertility carried a homozygous *DNAAF3* mutation (c.551T>A). The patient's wife carried the wild‐type sequence. The proband's parents and sister carried a heterozygous *DNAAF3* mutation. The red rectangle indicates the mutation site. (C) A schematic representation of *DNAAF3* exon structure and all mutation positions identified in the LOVD public variation database are marked. The red arrow indicates the new mutation position identified in the family. (D) Conservative analysis of the amino acids encoded by the biallelic mutation sites in different species.

Whole‐exome sequencing (WES) was performed to find the potential cause. In a previous study, homozygous mutations of *DNAAF3* gene were reported as the cause of PCD, including p.Leu108Pro, p.Arg136X and p.Ala325Thr (Figure 3C). In this patient, we detected a novel mutation of *DNAAF3*: (NM_001256714) exon 5: c.551T>A: p.V184E. The pathogenic grade of this variant was assessed as suspected pathogenicity according to the ACMG guidelines. The mutation was not included in the dbSNP, 1000 Genomes, EXAC databases or NHLBI‐ESP polymorphism databases and was not found in normal control population. The p.V184E mutation was predicted with 91.9% confidence to be ‘damaging’ using Polyphen2 and SIFT. In addition, the phenotype of the variant carrier is highly consistent with the disease and is highly conserved across ciliated species (Figure 3D). Pedigree analysis showed that PCD with severe asthenozoospermia caused by *DNAAF3* mutation is an autosomal‐recessive mode of inheritance (Figure 3A). Sanger sequencing was used to confirm the homozygous mutation in *DNAAF3* in the patient and his family. Sanger sequencing results showed that his father and mother carried a heterozygous mutation in *DNAAF3* (Figure 3B). In addition, his unaffected sister also carried a heterozygous mutation (Figure 3B). Based on these results, we hypothesized that the homozygous mutation in *DNAAF3* was associated with PCD combined with severe asthenozoospermia.

### 3.5 The sperm flagella axonemal dynein arms were absent in the PCD patient with *DNAAF3* mutation

**FIGURE 4:**
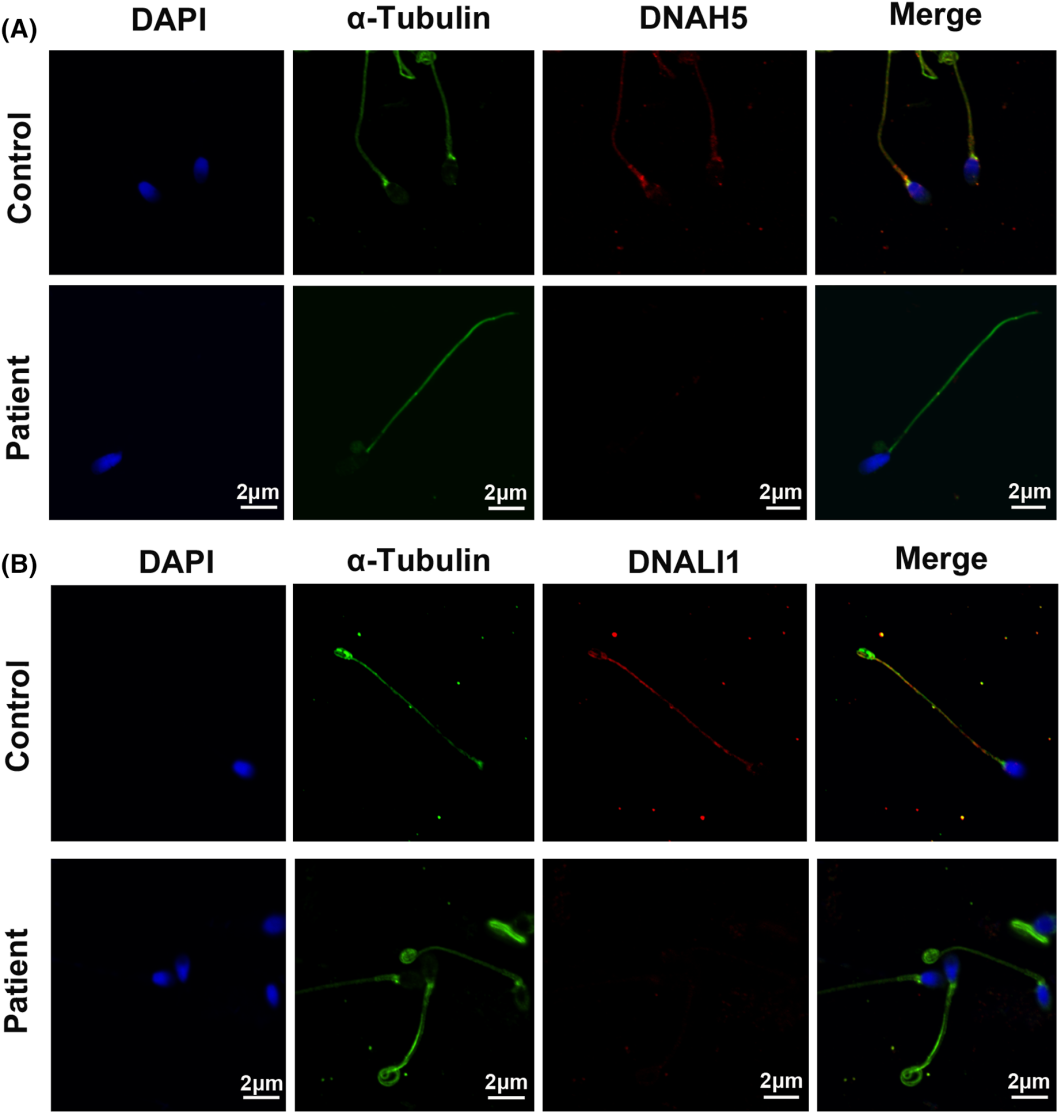
Immunofluorescence staining of spermatozoa from a control individual and the proband (II:1) with dynein arm protein antibody and α‐tubulin. Anti‐α‐tubulin was used to label the spermatozoa axoneme (green). Spermatozoa were counterstained with DAPI (blue) as a nuclei marker. (A) Outer dynein arm heavy chain protein DNAH5 (red) and α‐tubulin (green) localized along the entire sperm from the control, but complete absence of DNAH5 from the spermatozoa axoneme from the proband. (B) Inner dynein arm light intermediate chain protein DNALI1 (red) was detected along the entire sperm with α‐tubulin (green) from the control, but was partly detected in the spermatozoa axoneme from the proband. Scale bars: 2 μm.

Since DNAH5 and DNALI1 have been used to detect the ODA heavy chain protein and the IDA light intermediate chain protein of sperm in previous studies^20^, ^30^ and the disruptions in DNAH5 are associated with male infertility,^20^, ^31^ we chose DNAH5 and DNALI1 as detective objective of ODA heavy chain protein and the IDA light intermediate chain protein of sperm. In order to further confirm that the *DNAAF3* mutation leads to flagella axonemal dynein defects in PCD patient with severe asthenozoospermia, we analysed the presence and location of ODA heavy chain protein DNAH5 and IDA light intermediate chain protein DNALI1 in spermatozoa from a control and the PCD patient with *DNAAF3* mutation by performing immunofluorescence analyses. DNAH5 and DNALI1 co‐expressed with the α‐tubulin along the sperm flagellum from the control (Figure 4A,B). However, DNAH5 was not detected and DNALI1 was partly detected along the full length of the flagellum in the PCD patient with *DNAAF3* mutation (Figure 4A,B), which is consistent with the absence of ODAs and IDAs of TEM results. Taken together, outer dynein arms (ODAs) and inner dynein arms (IDAs) were all affected by *DANAF3* mutation due to the impaired assembly of dynein arm complexes.

### 3.6 Effects of *DNAAF3* mutation on its expression

**FIGURE 5:**
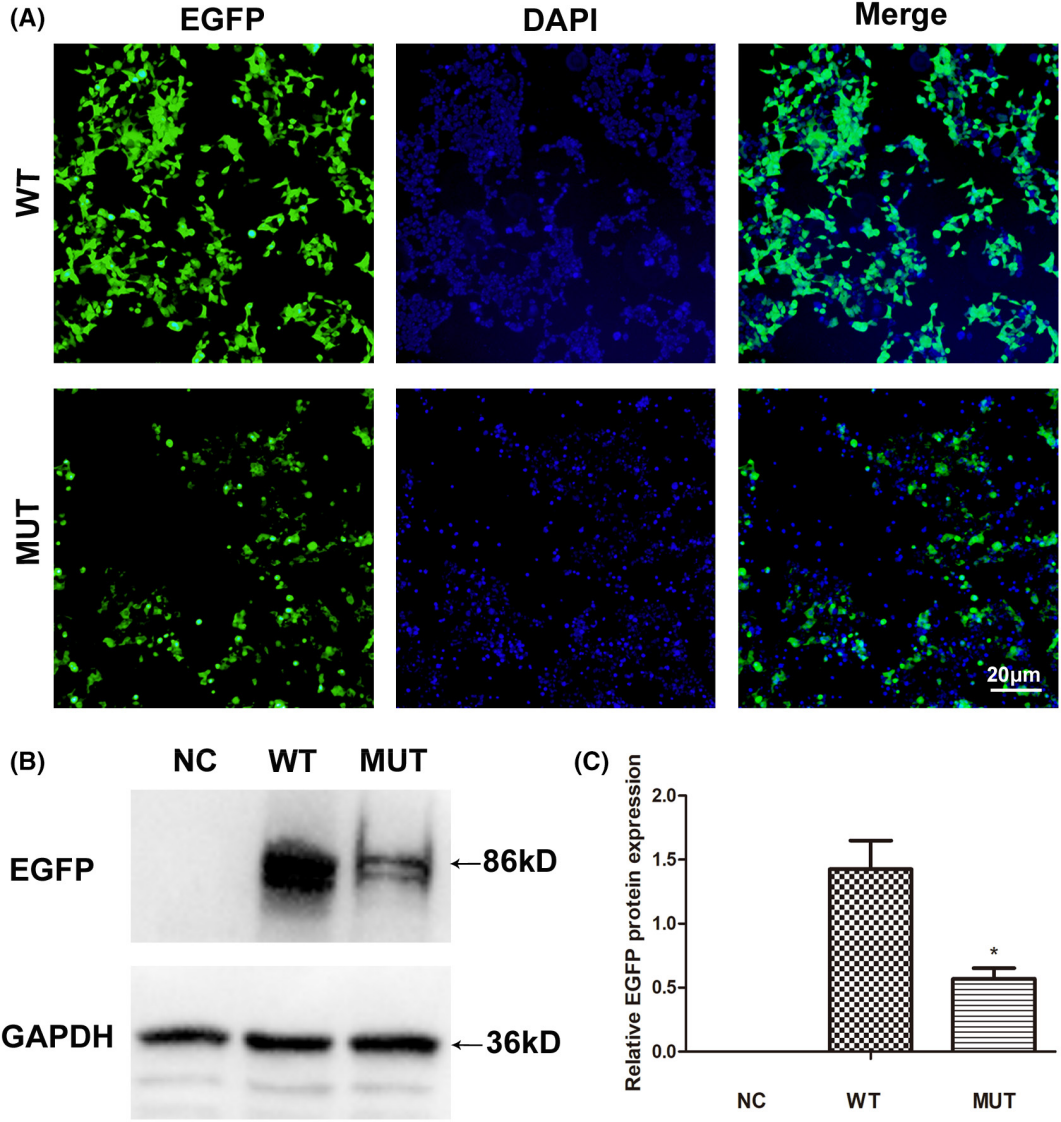
Effects of *DNAAF3* mutation on protein expression in vitro. (A) Wild‐type and mutant‐type *DNAAF3* plasmids were separately transfected into the 293T cells to observe the EGFP fluorescence of DNAAF3‐EGFP fusion protein. (B) The expression levels and size of EGFP protein in different groups were detected by western blot. The protein molecular weight represents the fusion protein expression of DNAAF3 and EGFP. (C) The EGFP protein expression difference between groups was counted and analysed based on the grey values of the detected bands in (B). ‘*’ indicated statistically significant difference (*p* < 0.05). EGFP, enhanced green fluorescent protein; NC, cells transfected with lipofectamine; WT, cells transfected with wild‐type *DNAAF3* plasmid. MUT, cells transfected with mutated‐*DNAAF3* plasmid (c.551T>A).

To analyse the effects of *DNAAF3* mutation on protein expression, we performed a transient transfection assay with the wild‐type (WT) and mutant‐type (MUT) *DNAAF3* (c.551T>A) expression vectors and transfected them into the HEK‐293 T cells. Twenty‐four hours after transfection, the EGFP fluorescence of mutant‐type *DNAAF3* group was weaker than that of wild‐type *DNAAF3* group (Figure 5A). Since DNAAF3 was fused with the EGFP tag as DNAAF3‐EGFP fusion protein, the expression of EGFP proteins reflects the expression levels of DNAAF3 protein. Seventy‐two hours after transfection, western blot was performed to detect EGFP protein levels in different groups of cells. The results showed that the expression of mutant DNAAF3 protein was significantly decreased compared with that of the wild type (Figure 5B,C).

### 3.7 Intracytoplasmic sperm injection with the patient's sperm and pregnancy outcome

Due to the reduction or absence of motile sperms, ICSI is the major choice for PCD patients to obtain healthy offspring. Thirty‐eggs were retrieved after the long‐acting GnRHa protocol, and 24 mature eggs were chosen for ICSI procedure from the partner of the patient. Seventeen embryos developed into two‐cell stage by Day 1. All the two‐cell stage and eight‐cell stage embryos were further cultured. Two and four embryos developed into blastocyst stage by Day 5 and Day 6, respectively, and all the good‐quality blastocyst stage embryos were frozen because of personal factors. One frozen embryo was transferred into his wife's uterine under ultrasound guidance 2 months later. The partner of the patient became pregnant, and one healthy baby was born.

## 4 DISCUSSION

DNAAF3 has been reported to be associated with PCD due to its essential role in dynein assembly of ciliary axoneme. A previous study detected three different mutations of *DNAAF3* in PCD individuals: c.323T>C (p.Leu108Pro) in exon 3 creating missense mutation predicted to be ‘probably damaging’, c.406C>T (p.Arg136X) in exon 4 creating nonsense mutation, and c.762_763insT in exon 6 (p.Val255CysfsX12), creating a predicted frameshift that generates 11 novel amino acids after a p.Val255Cys change followed by a premature stop codon.^27^

Although sperm flagella and motile cilia share a highly similar axonemal structure, it remains unknown whether *DNAAF3* mutation in PCD patients affect male fertility due to impaired sperm motility with loss of the flagella dynein arm. A previous study found a case of male infertility due to immobility sperm in PCD patient with *DNAAF3* mutations, but did not investigate the fertility status of other PCD patients. In addition, the sperm ultrastructure and outer and inner dynein arms (ODA/IDA) from the infertility patient were not detected.^27^ In this study, we identified a novel biallelic mutation in the *DNAAF3* gene (c.551T>A; p.V184E) in a PCD patient with infertility due to severe asthenozoospermia. TEM and immunostaining analysis showed axoneme defects with loss of both outer and inner dynein arms in the spermatozoa of the PCD patient. These findings revealed that the PCD‐associated gene *DNAAF3* was linked to male infertility because of impaired spermatozoa motility.

The proper axonemal dyneins assembly process is a multi‐step pathway that contains cytoplasmic assembly of axonemal dyneins that may involve in proper folding/stability of individual DHCs as well as their association into larger, multi‐subunit complexes and the dynein complexes assembling into flagella.^27^ DNAAF3, also known as PCD, DAB1, PF22, CILD2 and C19orf51, is required for the assembly of axonemal inner and outer dynein arms and subsequent transport into cilia, where it works with a chaperone complex comprised of other characterized assembly factors, like PF12/DNAAF1 and ODA7/DNAAF2.^24^, ^25^, ^26^ However, function of PF22/DNAAF3 appears different from that of other previously identified PCD proteins like PF13/DNAAF1 and ODA7/DNAAF2. Both PF13 and ODA7 may function in aid folding dynein heavy chains (DHCs). Different from the role in folding globular head domains, PF22 may function in joining other subunits of heavy chains and smaller subunits into a larger complex as a co‐chaperone and may be required for dissociation of the chaperone complex at the completion of assembly. Loss of function of PF22/DNAAF3 in Chlamydomonas impairs the normal assembly of ODA complex into flagella and formation of functional dynein arms, suggesting that the failure of ODA complex assembly involve in the IFT transport machinery or binding sites on the axoneme's peripheral doublet microtubules. In zebrafish, *DNAAF3* knockdown disrupts dynein assembly and abolishes motility of cilia, resulting in left–right axis patterning defect of embryo like PCD patients, suggesting that DNAAF3 may play an important role in cytoplasmic preassembly of axonemal dyneins for cilia motility.^24^, ^32^, ^33^ Further research found that mutations in *DNAAF3* block assembly of DNALI1 and lead to the residual DNAH5 and DNALI1 in the apical cytoplasm in some PCD patients.^27^ We identified a novel biallelic mutation in *DNAAF3* gene (c.551T>A; p.V184E) in a PCD patient with infertility with severe asthenozoospermia. TEM found the ultrastructural axoneme defects with loss of both outer and inner dynein arms. Immunostaining analysis further found that the DNAH5 and DNALI1 proteins were absent in the spermatozoa of the PCD patient. In addition, in vitro functional experiment, western blot assay results showed that the mutation of *DNAAF3* caused a significant decrease in the DNAAF3 protein, which indicating that the mutation of *DNAAF3* might damage the DNAAF3 protein. These findings revealed that the novel *DNAAF3* mutation may also disrupt the assembly of dynein arms and affect the motility of spermatozoa due to the loss of both outer and inner dynein arms. However, due to the limitations of sample size, it is necessary to test *DNAAF3* variants in a larger cohort of patients to reveal the effects of DNAAF3 on the motility of spermatozoa in PCD patients and to further confirm the role of the *DNAAF3* mutation in severe asthenozoospermia in the future study. In addition, due to species differences among Chlamydomonas, zebrafish and mammals, the phenotype of *DNAAF3* mutations may be different. Thus, we will generate *dnaaf3* knockout mice using CRISPR/Cas9 technology to further validate the role for DNAAF3 in assembly of ODA/IDA protein, such as DNAH5 and DNALI1, in spermatogenesis.

## AUTHOR CONTRIBUTIONS

**Feng Wan:** Conceptualization (equal); supervision (equal); visualization (equal); writing – original draft (equal). **Lan Yu:** Investigation (equal); project administration (equal); resources (equal). **Xiaowei Qu:** Data curation (equal); methodology (equal); resources (equal). **Yanqing Xia:** Methodology (equal); resources (equal). **Ke Feng:** Methodology (equal); resources (equal). **Lei Zhang:** Formal analysis (equal); software (equal); validation (equal); visualization (equal). **Na Zhang:** Data curation (equal); investigation (equal); resources (equal). **Guihua Zhao:** Data curation (equal); resources (equal). **Cuilian Zhang:** Writing – review and editing (equal). **Haibin Guo:** Funding acquisition (equal); supervision (equal); validation (equal); writing – review and editing (equal).

## CONFLICT OF INTEREST STATEMENT

The authors declare that they have no conflict of interest.

## Data Availability

The data that support the findings are available from the corresponding author upon reasonable request.
